# Supplementary material for: Isolation of pseudocapacitive surface processes at monolayer MXene flakes reveals delocalized charging mechanism
Source: Nat Commun. 2023 Jan 23;14:374. doi: 10.1038/s41467-023-35950-1 (PMC9870982; doi:10.1038/s41467-023-35950-1)
Supplement: Supplementary file 1 — Supplementary Information [file 41467_2023_35950_MOESM1_ESM.pdf]

Isolation of pseudocapacitive surface processes at monolayer MXene flakes reveals delocalized charging mechanism

Marc Brunet Cabré,[1] Dahnan Spurling,[1] Pietro Martinuz,[1,2] Mariangela Longhi,[2] Christian Schröder,[1] Hugo Nolan,[1] Valeria Nicolosi,[1] Paula E. Colavita,[1] Kim McKelvey[1,3*]

[1] School of Chemistry, Trinity College Dublin, Dublin 2, Ireland.

[2] Università degli Studi di Milano, Dipartimento di Chimica, Via Golgi 19, 20133 Milano, Italy.

[3] MacDiarmid Institute for Advanced Materials and Nanotechnology, School of Chemical and Physical Sciences, Victoria University of Wellington, Wellington 6012, New Zealand.

*kim.mckelvey@vuw.ac.nz

Inventory of supporting information

Supplementary Note 1. Structural and morphological characterization of $Ti_3C_2T_x$ **3**

Supplementary Fig. 1: X-ray diffractogram (XRD) of $Ti_3C_2T_x$ freestanding films. 3

Supplementary Fig. 2: Energy dispersive X-ray spectroscopy (EDX) of $Ti_3C_2T_x$ freestanding films. 3

Supplementary Fig. 3: Raman spectra of $Ti_3C_2T_x$ freestanding films. 4

Supplementary Fig. 4: Optical image of substrate with $Ti_3C_2T_x$ drop-cast. 4

Supplementary Fig. 5: AFM characterization for MXene flakes. 5

Supplementary Table 1: Step-heights obtained from AFM images. 6

Supplementary Fig. 6: SEM micrographs obtained after SECCM scans. 7

Supplementary Table 2: Area of $Ti_3C_2T_x$ flakes as determined from SEM micrographs. 7

Supplementary Note 2. Local electrochemical measurements **8**

Supplementary Table 3: Number of SECCM points examined and classified according to the surface morphology probed. 8

Supplementary Fig. 7: Assignation of each point a surface morphology type based on droplet cell residue observed on SEM images. 9

Supplementary Fig. 8: Distribution of active geometric areas obtained from SEM images of electrolyte residues after SECCM. 10

Supplementary Fig. 9: First loop of cyclic voltammograms 11

Supplementary Fig. 10: Second loop cyclic voltammograms 12

Supplementary Fig. 11: Second loop cyclic voltammograms on multilayer. 13

Supplementary Note 3: Specific capacitance calculations **14**

Supplementary Table 4: Area of $Ti_3C_2T_x$ flakes as determined from SEM micrographs and equivalent mass of each flake used to obtain data reported in the main text. 15

Supplementary Table 5: Summary of capacitance values reported in literature $Ti_3C_2T_x$ electrodes. 15

Supplementary Note 4: Ambient conditions **16**

Supplementary Fig. 12: Ambient conditions inside the faradaic cage where AFM and SECCM measurement were conducted. 16

References **17**

Supplementary Note 1. Structural and morphological characterization of $Ti_3C_2T_x$

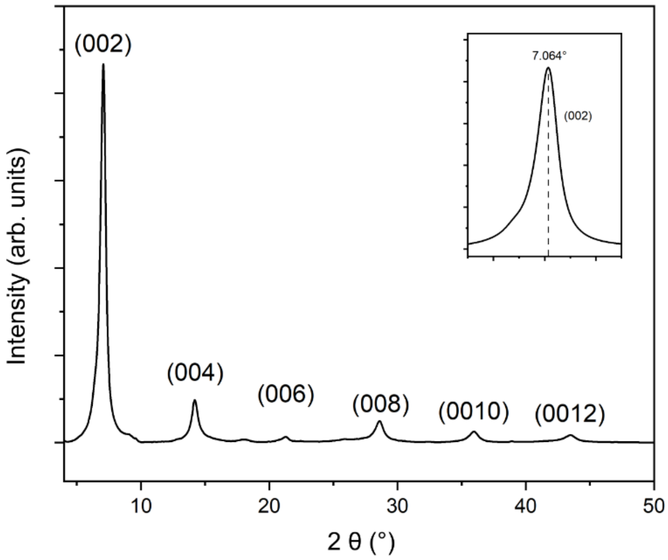

Supplementary Fig. 1: X-ray diffractogram (XRD) of $Ti_3C_2T_x$ freestanding films. Diffractogram shows the characteristic peak (0 0 2) at 7.064° and its reflections up to (0 0 12).[1] No trace of an anatase (TiO_2) peak at around 2θ = 25° is observed.

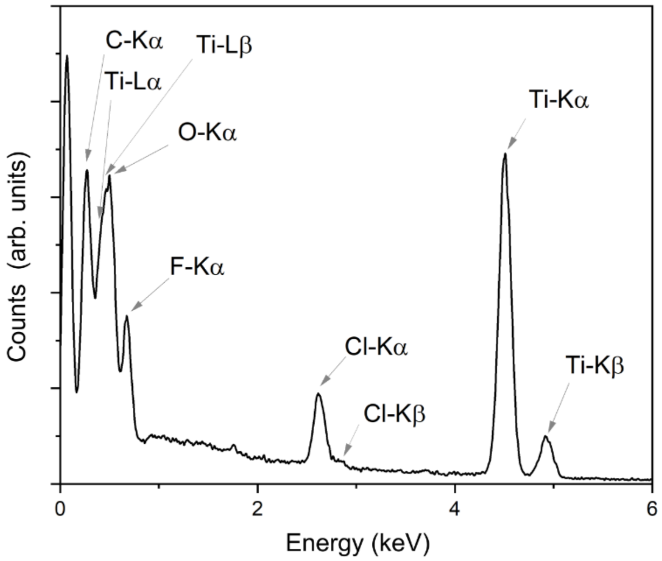

Supplementary Fig. 2: Energy dispersive X-ray spectroscopy (EDX) of $Ti_3C_2T_x$ freestanding films. Spectra shows the presence of the surface group atoms. No Al trace is observed suggesting complete MXene exfoliation. Halogen elements (F, Cl) observed are introduced during the exfoliation of MAX phase with LiF and HCl solution.[1]

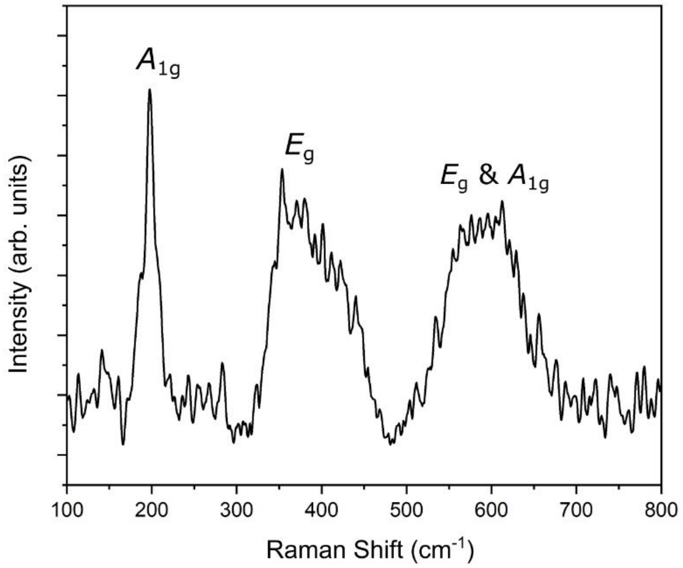

Supplementary Fig. 3: Raman spectra of $Ti_3C_2T_x$ freestanding films. Spectra shows characteristic peaks: A_{1g} at 200 cm^{-1}, E_g within the 230 – 470 cm^{-1} region and both E_g and A_{1g} in the 580 - 730 cm^{-1} region. [2]

Optical images, as shown in Supplementary Fig. 4, were used to locate $Ti_3C_2T_x$ flakes. SECCM measurements and further AFM and SEM characterization were performed over those regions.

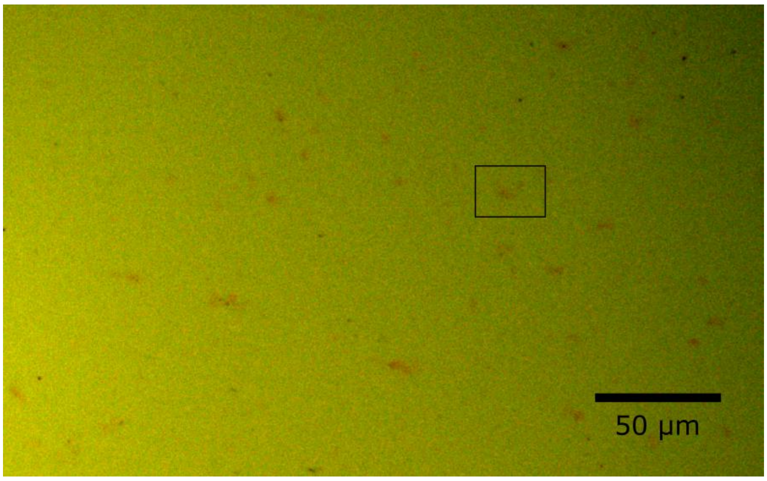

Supplementary Fig. 4: Optical image of substrate with $Ti_3C_2T_x$ drop-cast. Black square indicates the region of sample where experiments were conducted.

The AFM image in Supplementary Fig. 5A shows four distinct MXene flakes supported on the carbon thin film electrode; the circular features distributed in a square grid correspond to inorganic residues left by the SECCM droplet cell. Supplementary Fig. 5B shows four height profiles measured at step edges between the carbon substrate and the basal plane of $Ti_3C_2T_x$ flakes, corresponding to four different flakes, as indicated using solid lines in Supplementary Fig. 5A. The average step-height was found to be 3.4 ± 0.4 nm (see Supplementary Table 1). The $Ti_3C_2T_x$ monolayer thickness obtained by TEM studies and DFT calculations is estimated to be 0.98 nm;[3,4,5] however, experimental AFM step

heights in the range 2-3 nm are typically reported for $Ti_3C_2T_x$ monolayers supported on SiO_2.[6,7,8,9] These larger values of step-height observed via AFM are commonly attributed to a combination of the nature of the substrate/2D material interface, presence of adsorbates on 2D materials and possible instrumental factors.[9,10,11] Adsorbed water layers and/or water inter-layers between monolayer and substrate, are a characteristic of deposited $Ti_3C_2T_x$ monolayers, as is also the case for other 2D materials discussed in the literature.[12,13,14] Furthermore, a recent study observed that AFM step-height profiles of $Ti_3C_2T_x$ monolayers vary depending on the exfoliation procedure used, because differences in the exfoliation process can result in different surface functionalities, leading to variable densities of adsorbed water at their surface.[8] Therefore, AFM thickness determinations of $Ti_3C_2T_x$ monolayers are highly dependent on the presence of adsorbates and on the specific substrate of choice. Based on the combined AFM and SEM characterization, the step-height of 3.4 ± 0.4 nm measured between carbon and the MXene flake, can be attributed to a monolayer of $Ti_3C_2T_x$ with the presence of a water adlayer and, likely, also of an interlayer trapped between the flake and the carbon substrate.

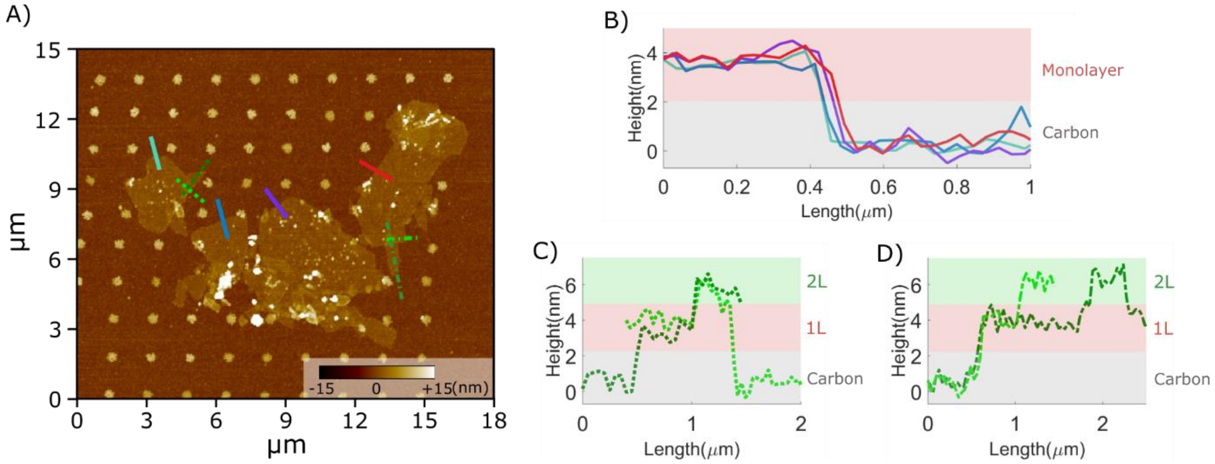

Supplementary Fig. 5: AFM characterization for MXene flakes. AFM image carried out after SECCM. **A**. AFM map of MXene flakes, also showing left over SECCM droplet residues. Solid coloured lines indicate region where step profiles from carbon to monolayer was taken. Doted lines indicate regions where step profiles from carbon to monolayer to bilayer where taken. **B.** Step profiles from carbon surface to monolayer MXene surface. **C**. and **D.** Step profiles from carbon surface to monolayer surface and to bilayer surface.

Supplementary Fig. 5A shows that some of the monolayer flakes are immobilized overlapping each other, resulting in bilayer regions. Height profiles from carbon to monolayer and to bilayer were acquired in regions indicated in Supplementary Fig. 5A by a dotted line and displayed in Supplementary Fig. 5C and 5D. The step-height from monolayer to bilayer was found to be 2.28 ± 0.63 nm (see Supplementary Table 1), considerably smaller than step-height values obtained for carbon (substrate) to monolayer. This is in good agreement with observations from other studies, where reported step-height values for monolayer to bilayer are considerably smaller than for substrate to monolayer.[8]

Supplementary Table 1: Step-heights obtained from AFM images.

$Ti_3C_2T_x$ Morphology		Step-height (nm)	Standard deviation (nm)
Carbon to monolayer MXene	Profile 1	3.35	0.29
	Profile 2	3.35	0.29
	Profile 3	3.59	0.38
	Profile 4	3.39	0.32
	Mean	3.42	0.35
Monolayer to bilayer MXene	Profile 1	1.85	0.48
	Profile 2	2.47	0.61
	Profile 3	2.22	0.50
	Profile 4	2.59	0.55
	Mean	2.28	0.63

SEM imaging was carried out using the In-Lens and the secondary electron (SE2) detectors. The In-Lens detector, see Supplementary Fig. 6A, clearly resolves the locations of electrolyte residues on the sample. This enabled us to assign the composition of the surface at each SECCM measurement point, and its classification as being on either carbon, or $Ti_3C_2T_x$ flakes, as detailed in Supplementary Fig. 7. In Supplementary Fig. 6B, the secondary electron detector (SE2) provided clear contrast that allowed us to determine the number of $Ti_3C_2T_x$ layers stacked on the substrate, with areas with overlapping flakes being clearly differentiated as brighter regions. Four isolated flakes of area larger than 5 μm^2 were identified in Supplementary Fig. 6B, as also shown in Figure 6A, and their areas are reported in Supplementary Table 2. The four larger flakes and most of the smaller flakes consist of the same number of layers. Given that the exfoliation method used achieves a monolayer yield of 80%,[15] the secondary electron SEM micrograph corroborates assignment of the four largest $Ti_3C_2T_x$ flakes as monolayer.

A)

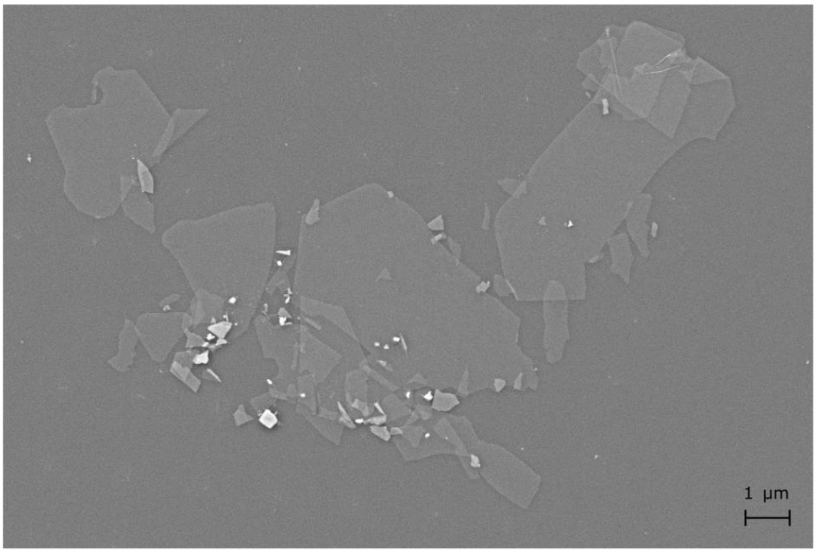

B)

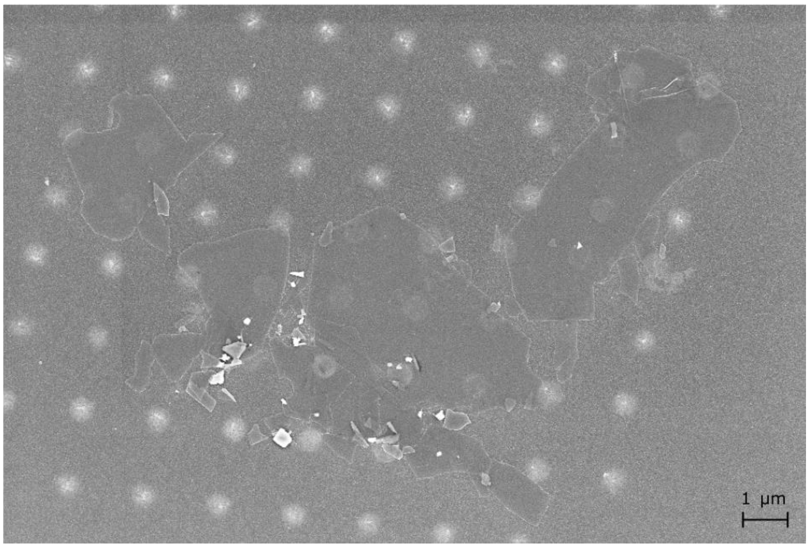

Supplementary Fig. 6: SEM micrographs obtained after SECCM scans. **A.** Image obtained with the SEM In-Lens detector. **B**. Image obtained with the SEM secondary electron detector (SE2).

Supplementary Table 2: Area of $Ti_3C_2T_x$ flakes as determined from SEM micrographs.

Related with Figure 6A in the main text.

Flake label	Flake size (μm^2)
(i)	5.87 ± 0.03
(ii)	5.13 ± 0.03
(iii)	15.43 ± 0.09
(iv)	14.14 ± 0.04

Supplementary Note 2. Local electrochemical measurements

Electrolyte residues are a common feature of SECCM mapping that enable identification of the contacted areas probed by the SECCM droplet cell.[16] The relative position of residue points can therefore be correlated with the surface morphology probed in each case. Supplementary Fig. 7A identifies a total of 80 points in the SECCM mapping grid that are visible over the area imaged in the SEM micrograph; of these, 67 points present a well-defined circular geometry that is suitable for area normalization of current densities, while the remaining 13 points are indicated with crosses in Supplementary Fig. 7B. On the basis of their locations in the SEM micrograph, the electrochemical responses of these 67 points were each assigned to that of either the carbon substrate (40 points) or the $Ti_3C_2T_x$ MXene (24 points). For the remaining 3 points it is not possible to unambiguously establish if the droplet cell was in contact with the MXene flake, therefore these 3 points were excluded from further analysis. The SE2 detector contrast further enabled to discriminate whether contact to $Ti_3C_2T_x$ was established over a monolayer basal plane exclusively (5 points, in red), at the edge of a monolayer (7 points, in blue), or over a multilayer region (12 points, in green). The classification of all 64 points according to the above four morphologies is shown in Supplementary Fig. 7C, and a summary of the number of points for each category is reported in Supplementary Table 3.

Supplementary Table 3: Number of SECCM points examined and classified according to the surface morphology probed.

	Number of Points
Points visualized in SEM micrograph	80
Points with well-defined geometric area	67
Points with well-defined surface contact	64
Surface morphology contacted	
$Ti_3C_2T_x$ - Monolayer basal-plane	5
$Ti_3C_2T_x$ - Carbon and Monolayer	7
$Ti_3C_2T_x$ - Multilayer Stack	12
$Ti_3C_2T_x$ - Total	24
Carbon	40

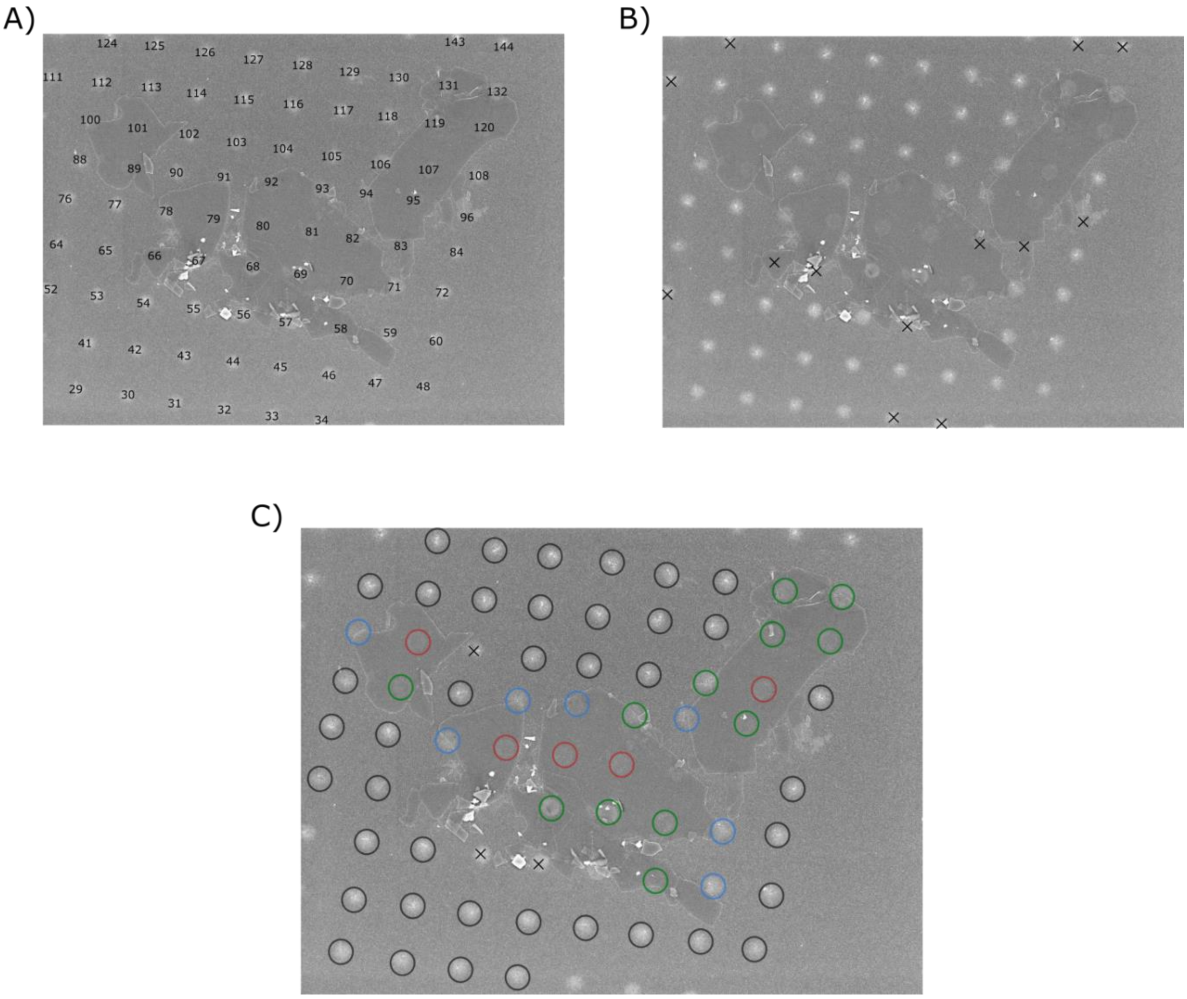

Supplementary Fig. 7: Assignation of each point a surface morphology type based on droplet cell residue observed on SEM images. **A.** Identification of all SECCM grid points visualized in the SEM micrographs; each point indicated with corresponding numbered position in the grid. **B.** Identification of points without well-defined circular geometric area (black cross). **C.** Classification of points with well-defined circular geometric area and non-ambiguous contact respect the surface contacted; only carbon contact (black circle), only monolayer basal-plane contact (red circle), partial carbon and partial monolayer contact (blue circle) and partial or complete multilayer contact (green circle). For three points (black crosses) it is ambiguous if they contact only carbon or partial carbon and partial monolayer contact.

The electrochemically active geometric area was estimated from the diameter of each residue in the SEM images and Supplementary Fig. 8A shows the distribution of values thus obtained. The average area contacted by the droplet cell was found to be 0.31 ± 0.02 μm^2. The well-defined circular geometry observed for the majority of salt residues and the narrow distribution of electrochemical surface area values confirm that the SECCM droplet cell maintains a regular shape and does not spread over the contacted surface during measurements. Supplementary Fig. 8B shows the distributions of geometric area observed for carbon and for $Ti_3C_2T_x$ regions; the distributions display maxima at similar positions and mean values which are within their errors, indicating consistent droplet sizes at the two surfaces.

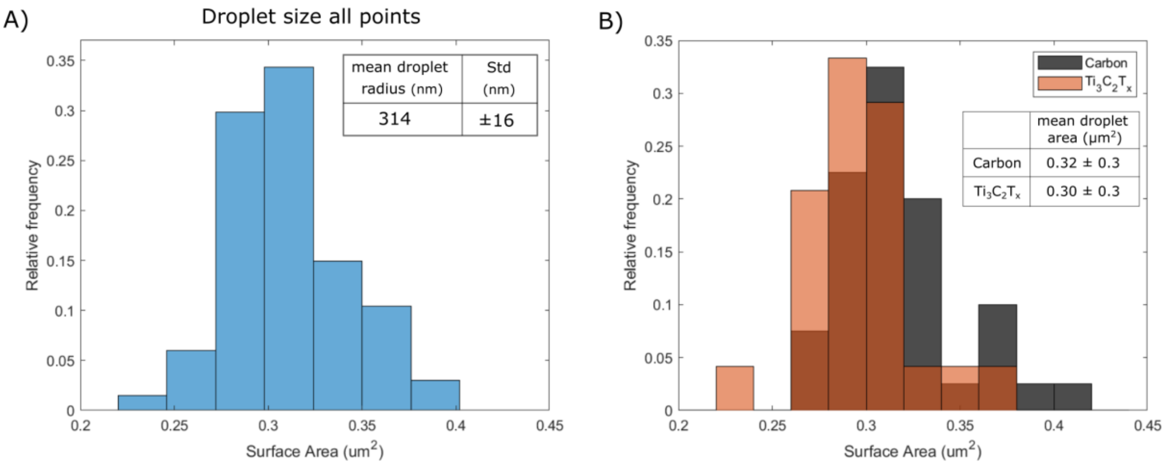

Supplementary Fig. 8: Distribution of active geometric areas obtained from SEM images of electrolyte residues after SECCM. **A.** Distribution of area values for all points with well-defined circular shape (N = 67). **B.** Overlap histogram showing distributions of droplet area values for points that correspond to carbon (N = 40, in black) and *$Ti_3C_2T_x$ (N = 24, in brown) regions.*

Cyclic voltammograms (CV) were measured at each probed point, between +0.500 V and -1.000 V vs Pd-H_2 at 0.500 V/s; two cycles were recorded in all cases. Supplementary Fig. 9 and 10 show the 1st and 2nd cycle, respectively, obtained on all carbon, mixed carbon/monolayer, and monolayer MXene sample points (for details of point assignment see Supplementary Table 3 and Supplementary Fig. 7). Differences can be observed when comparing the two cycles obtained at points corresponding to complete or partial probing of MXene flakes. These differences are common in the electrochemical response of MXenes and are attributed to conditioning during the initial cycle. [17] Therefore, the 2nd cycle was used for further analysis in all cases. As observed in Supplementary Fig. 9A and 10A, when only carbon is contacted the first and second cycle present an identical response on the pseudocapacitive regime (from 0.45V to -0.55V vs SHE). Supplementary Fig. 11 show the voltammograms obtained on points compose with partial or complete multilayer contact, compared with monolayer response and differentiated for 1st and 2nd cycle.

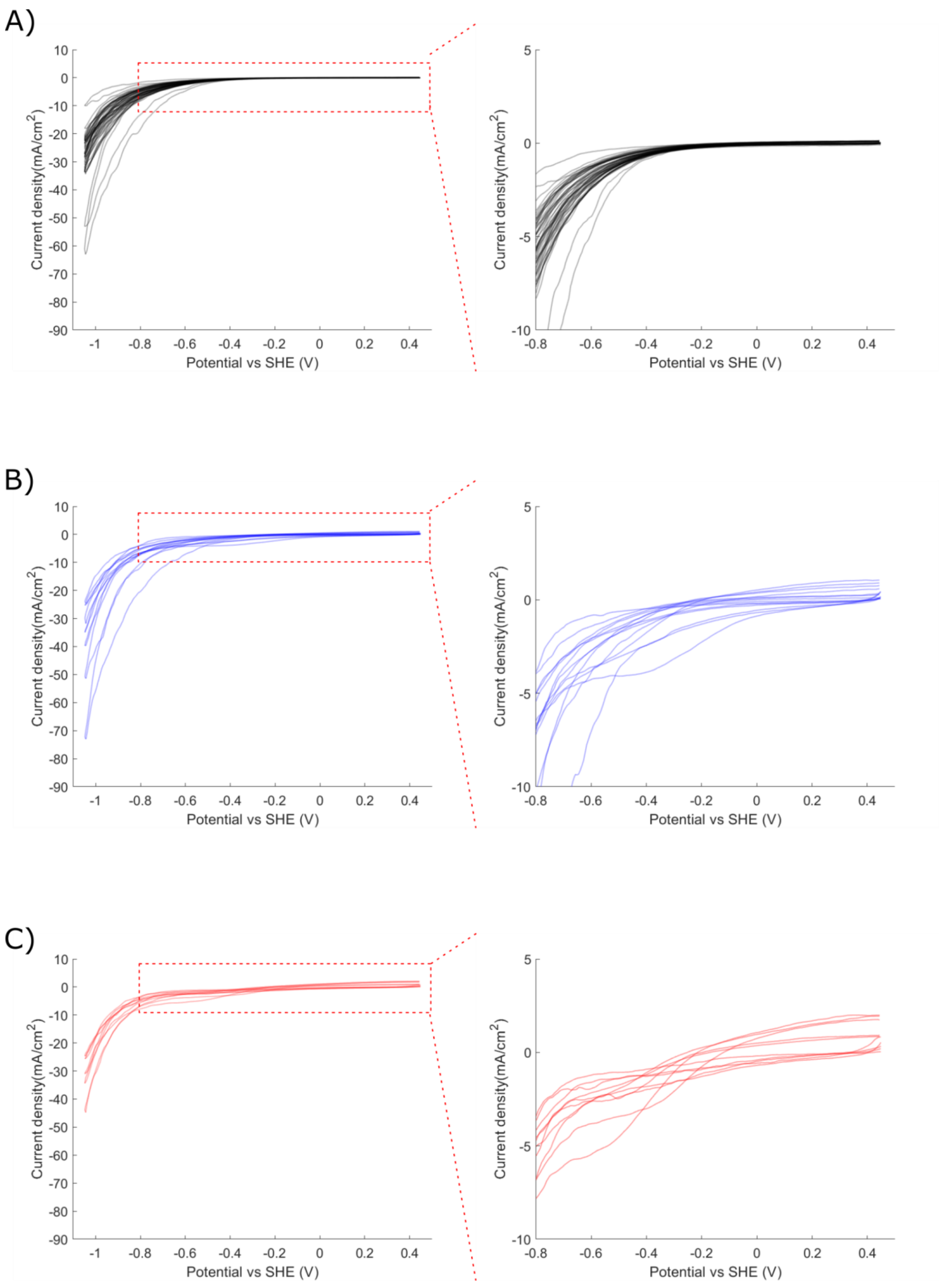

Supplementary Fig. 9: First loop of cyclic voltammograms on **A**. Carbon sample points, **B**. Mixed monolayer MXene and Carbon sample points, and **C**. monolayer (basal-plane) MXene sample points.

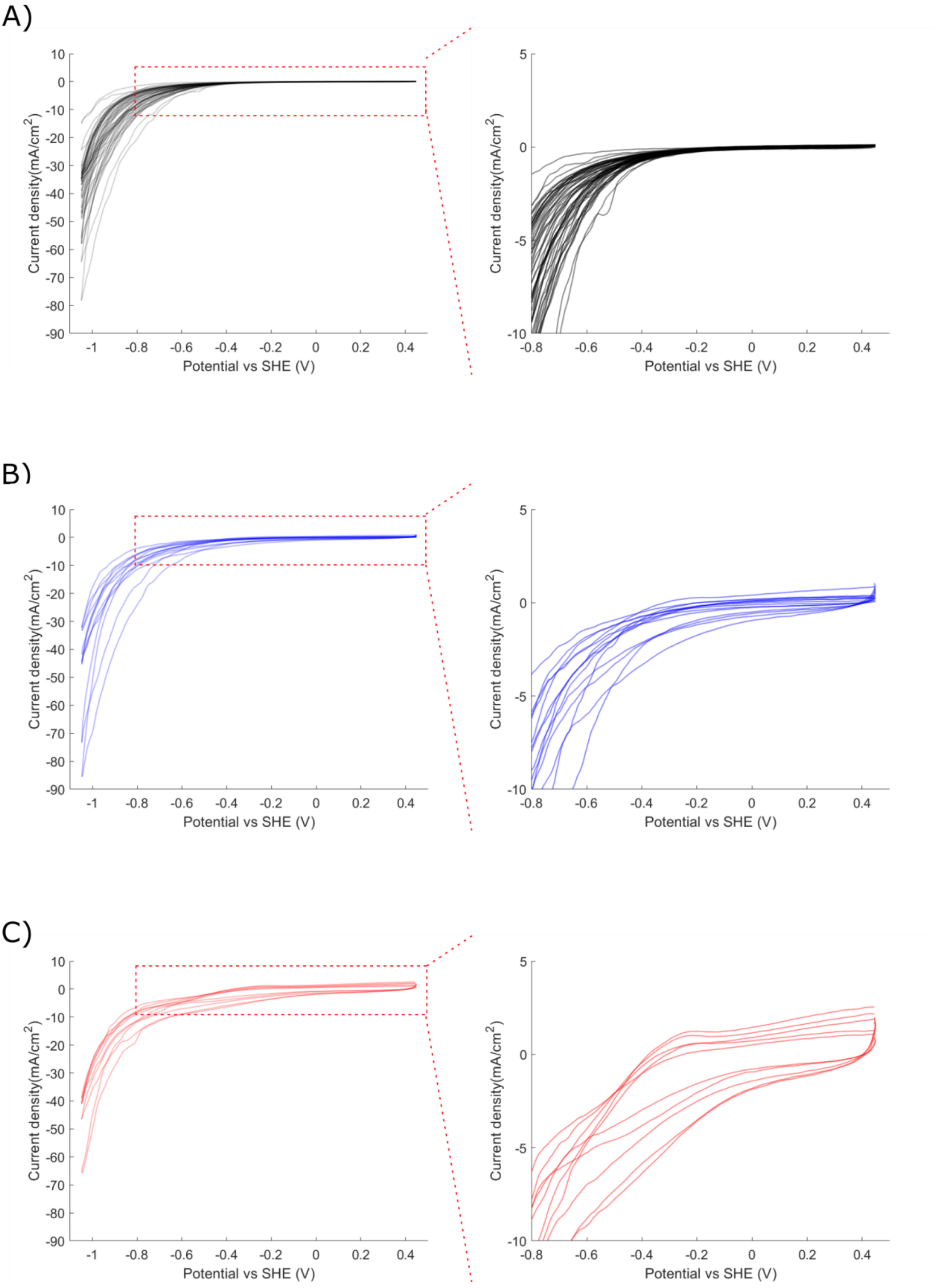

Supplementary Fig. 10: Second loop cyclic voltammograms on **A**. Carbon sample points, **B**. Mixed monolayer MXene and Carbon sample points, and **C**. monolayer (basal-plane) MXene sample points.

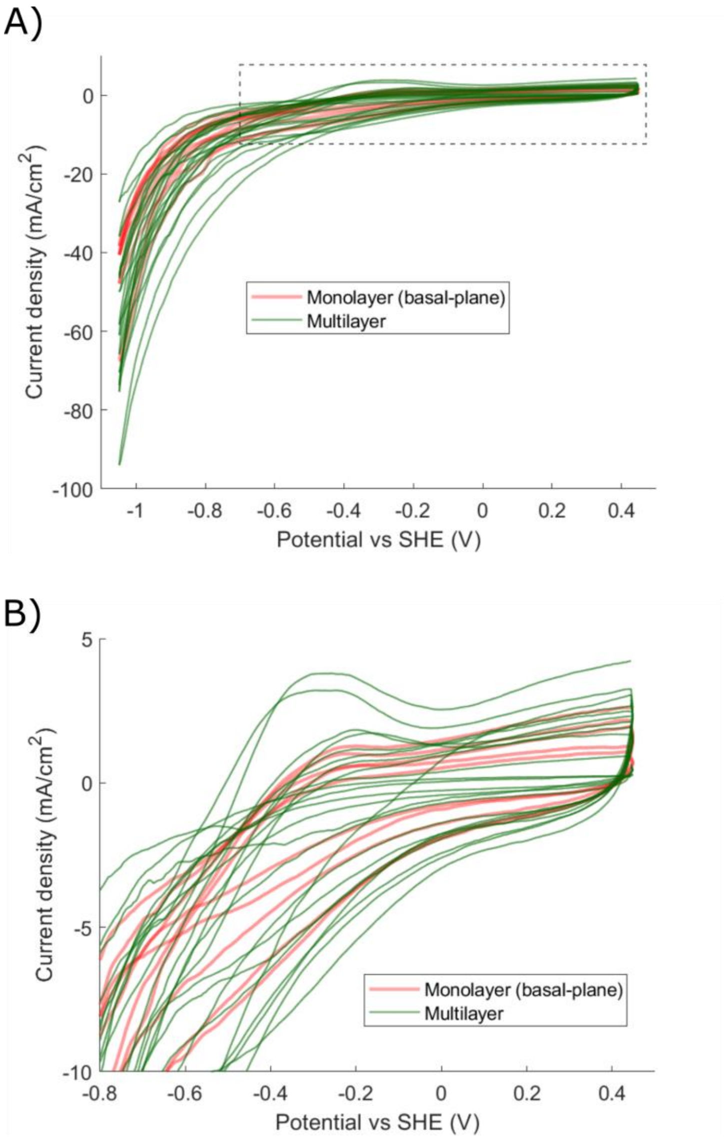

Supplementary Fig. 11: Second loop cyclic voltammograms on multilayer. **A.** SECCM points contacting multilayer flakes (> 2 layers) in green compared to the monolayer basal-plane response in red. Dotted square indicates magnified are for B. **B**. Maximize graph of region indicated in A. in dotted square profile.

Supplementary Note 3: Specific capacitance calculations

Specific gravimetric capacitance values were calculated based on the known crystal structure of $Ti_3C_2T_x$ monolayers, which consist of 12 unit cells/nm^2.[18] Thus, mass per unit area of $Ti_3C_2T_x$ monolayers was calculated as:

$$m = A * \frac{1}{\mathrm{SSA_{1L}}} \quad (1)$$

where m is the mass of $Ti_3C_2T_x$ under the droplet cell, A is the wetted area and SSA_{1L} is the specific surface area for $Ti_3C_2T_x$ monolayers. SSA_{1L} was calculated considering stoichiometry, molar mass and a surface termination, T_x, by –OH groups.

$$\frac{1}{\mathrm{SSA_{1L}}} = \frac{\text{mass of } \mathrm{Ti_3C_2OH}}{\mu\mathrm{m}^2 \text{ of monolayer}} = \frac{12 \text{ unit cells}}{\mathrm{nm}^2} \cdot \frac{10^6\ \mathrm{nm}^2}{1\ \mu\mathrm{m}^2} \, \frac{3{\cdot}\mathrm{Ti_{MW}} + 2{\cdot}\mathrm{C_{MW}} + 1{\cdot}\mathrm{O_{MW}} + 1{\cdot}\mathrm{H_{MW}}}{1 \text{ unit cell}} \cdot \frac{1}{\mathrm{N_A}} \quad (2)$$

$$\frac{1}{\mathrm{SSA_{1L}}} = 3.678 \cdot 10^{-15} \frac{\mathrm{g}}{\mu\mathrm{m}^2} \quad (3)$$

$$\mathrm{SSA_{1L-one\ side}} = 271.9 \frac{\mathrm{m}^2}{\mathrm{g}} \quad (4)$$

Specific surface capacitance values reported here were obtained on a flat 2D surface (i.e., the basal plane), and cannot be directly compared to the areal capacitance values of three-dimensional electrodes typically reported in literature. However, literature values of specific gravimetric capacitance for three-dimensional electrodes can be normalized by their specific surface area, in case provided, to derive their equivalent specific surface capacitance. Thus, it is possible to draw comparisons between reported capacitance values of three-dimensional electrodes and our measurements, as summarized in Supplementary Table 5.

SEM micrographs also allow for calculation of the size of flakes displayed in Figure 6A. By accounting the specific surface area for a $Ti_3C_2T_x$ monolayer (SSA_{1L}) the mass of each flake can be estimated, as shown in Supplementary Table 4.

Supplementary Table 4: Area of $Ti_3C_2T_x$ flakes as determined from SEM micrographs and equivalent mass of each flake used to obtain data reported in the main text.

Flake label	Flake size (μm^2)	Flake Mass (fg)
**(i)**	5.87 ± 0.03	21.5
**(ii)**	5.13 ± 0.03	18.9
**(iii)**	15.43 ± 0.09	56.8
**(iv)**	14.14 ± 0.04	52.0

Supplementary Table 5: Summary of capacitance values reported in literature $Ti_3C_2T_x$ electrodes.

		**Specific gravimetric capacitance (F/g)**	**Specific surface area (m^2/g)**	**Specific surface capacitance (mF/cm^2)**	**Reference**
**$Ti_3C_2T_x$ film**		231	16.2	1.43	19
**$Ti_3C_2T_x$ film**		220	19.2	1.145	20
**$Ti_3C_2T_x$ /Ni foam film**		350	32	1.093	21
**$Ti_3C_2T_x$ aerogel**		438	108	0.406	19
**$Ti_3C_2T_x$ hydrogel**		220	196	0.112	22
**3D printed $Ti_3C_2T_x$ aerogel**		242	177	0.137	23
**Monolayer $Ti_3C_2T_x$**	*Electrochemically contacted surface area*	*7700 ± 2800*	*SSA $_{1L\text{-one side}}$ = 271.9*	*2.8 ± 1.0*	This work
	Flake surface area	*240 ± 50*	*SSA $_{1L\text{- two sides}}$ = 543.9*	*0.04 ± 0.01*	

Supplementary Note 4: Ambient conditions

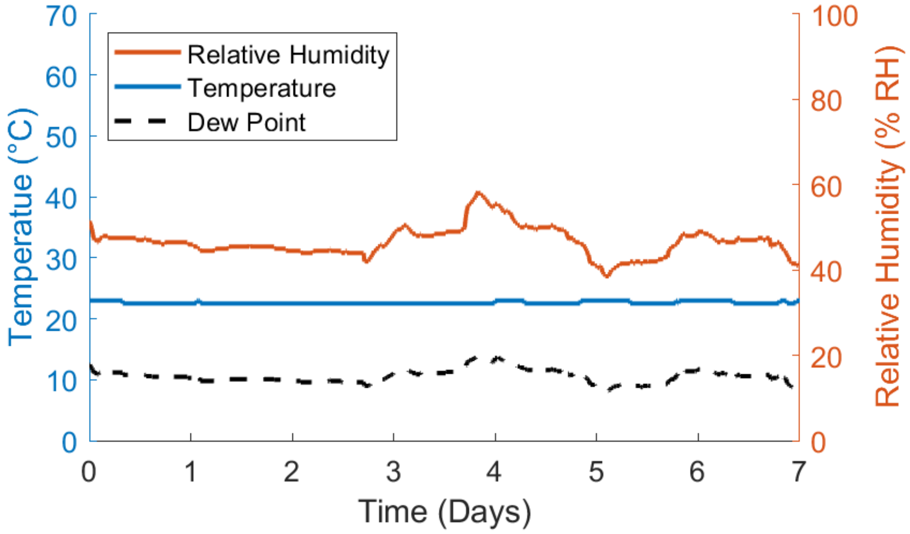

Supplementary Fig. 12: Ambient conditions inside the faradaic cage where AFM and SECCM measurement were conducted. Temperature (blue), humidity (orange) and dew point (black dotted line) recorded inside the faradic cage for 7 days. Temperature is very stable with mean value of 22.6 ± 0.2 °C. Humidity values oscillate between 40 – 60 % RH.

Supplementary References

1. Alhabeb, M. *et al.* Guidelines for Synthesis and Processing of Two-Dimensional Titanium Carbide (Ti 3 C 2 T x MXene). *Chem. Mater.* **29**, 7633–7644 (2017).

2. Sarycheva, A. & Gogotsi, Y. Raman Spectroscopy Analysis of the Structure and Surface Chemistry of Ti 3 C 2 T x MXene. *Chem. Mater.* **32**, 3480–3488 (2020).

3. Wang, X. *et al.* Atomic-scale recognition of surface structure and intercalation mechanism of Ti3C2X. *J. Am. Chem. Soc.* **137**, 2715–2721 (2015).

4. Ghidiu, M., Lukatskaya, M. R., Zhao, M. Q., Gogotsi, Y. & Barsoum, M. W. Conductive two-dimensional titanium carbide 'clay' with high volumetric capacitance. *Nature* **516**, 78–81 (2015).

5. Halim, J. *et al.* Transparent conductive two-dimensional titanium carbide epitaxial thin films. *Chem. Mater.* **26**, 2374–2381 (2014).

6. Cai, M. *et al.* Ti3C2Tx/PANI composites with tunable conductivity towards anticorrosion application. *Chem. Eng. J.* **410**, 128310 (2021).

7. Lipatov, A. *et al. Elastic properties of 2D Ti 3 C 2 T x MXene monolayers and bilayers*. *Sci. Adv.* **4**, 6 (2018).

8. Lipatov, A. *et al.* Effect of Synthesis on Quality, Electronic Properties and Environmental Stability of Individual Monolayer Ti3C2 MXene Flakes. *Adv. Electron. Mater.* **2**, 1600255 (2016).

9. Miranda, A., Halim, J., Lorke, A. & Barsoum, M. W. Rendering Ti3C2Tx (MXene) monolayers visible. *Mater. Res. Lett.* **5**, 322–328 (2017).

10. Mechler, Á. *et al.* Anomalies in nanostructure size measurements by AFM. *Phys Rev B Condens Matter Mater. Phys.* **72**, 1–6 (2005).

11. Shearer, C. J., Slattery, A. D., Stapleton, A. J., Shapter, J. G. & Gibson, C. T. Accurate thickness measurement of graphene. *Nanotechnology* **27**, 125704 (2016).

12. Mashtalir, O. *et al.* The effect of hydrazine intercalation on the structure and capacitance of 2D titanium carbide (MXene). *Nanoscale* **8**, 9128–9133 (2016).

13. Ochedowski, O., Bussmann, B. K. & Schleberger, M. Graphene on Mica - Intercalated Water Trapped for Life. *Sci. Rep.* **4**, 6003 (2015).

14. Coy Diaz, H., Addou, R. & Batzill, M. Interface properties of CVD grown graphene transferred onto MoS_2 (0001). *Nanoscale* **6**, 1071–1078 (2014).

15. Zhang, C. (John) *et al.* Additive-free MXene inks and direct printing of micro-supercapacitors. *Nat. Commun.* **10**, 1–9 (2019).

16. Ġuell, A. G., Lai, S. C. S., McKelvey, K., Snowden, M. E. & Unwin, P. R. Scanning electrochemical cell microscopy: A versatile technique for nanoscale electrochemistry and functional imaging neil ebejer1. *Annu. Rev. Anal. Chem.* **6**, 329–351 (2013).

17. Shao, H. *et al.* Unraveling the charge storage mechanism of Ti3C2Tx mxene electrode in acidic electrolyte. *ACS Energy Lett.* **5**, 2873–2880 (2020).

18. Zhan, C. *et al.* Understanding the MXene Pseudocapacitance. *J. Phys. Chem. Lett.* **9**, 1223–1228 (2018).

19. Wang, X. *et al.* 3D Ti3C2T: X aerogels with enhanced surface area for high performance supercapacitors. *Nanoscale* **10**, 20828–20835 (2018).

20. Zhang, P. *et al.* In Situ Ice Template Approach to Fabricate 3D Flexible MXene Film-Based Electrode for High Performance Supercapacitors. *Adv. Funct. Mater.* **30**, 2000922 (2020).

21. Hu, M. *et al.* Self-assembled Ti3C2Tx MXene film with high gravimetric capacitance. *ChemComm* **51**, 13531–13533 (2015).

22. Shang, T. *et al.* 3D Macroscopic Architectures from Self-Assembled MXene Hydrogels. *Adv. Funct. Mater.* **29**, 1903960 (2019).

23. Yang, W. *et al.* 3D Printing of Freestanding MXene Architectures for Current-Collector-Free Supercapacitors. *Adv. Mater.* **31**, 1902725 (2019).
